# Acute Coronary Syndrome and Acute Abdomen Suspected for Type B Aortic Dissection in an Elderly Woman

**DOI:** 10.7759/cureus.59556

**Published:** 2024-05-02

**Authors:** Fatima Ahmed, Eman Hassan, Sreenivas Muthyala

**Affiliations:** 1 Acute Medicine, University Hospital Coventry and Warwickshire NHS Trust, Coventry, GBR; 2 Radiology, University Hospital Coventry and Warwickshire NHS Trust, Coventry, GBR

**Keywords:** acute abdomen, contrast-enhanced ct-abdomen & pelvis, chest x-ray (cx-ray), : acute coronary syndrome, type b aortic dissection

## Abstract

Being an uncommon and challenging disorder, acute aortic dissection (AAD) can have fatal outcomes in the event of missed diagnosis or treatment delay. AAD could easily be misdiagnosed, as symptoms usually mimic other common clinical syndromes showing up in Accident and Emergency (A&E), including acute coronary syndrome (ACS), pericarditis, pulmonary embolism, acute abdomen, musculoskeletal pain, as well as presenting as heart failure, stroke, syncope, and absent peripheral pulses.

We present a case of a 77-year-old female who presented to the medical decision unit with acute-onset chest, back, and abdominal pain that occurred on standing for six hours She was thought initially to have acute coronary syndrome based on electrocardiography (ECG) changes, troponin, a normal chest X-ray, and no blood pressure discrepancies in upper extremities. Due to worsening abdominal pain and a previous history of a perforated diverticulum, contrast computed tomography (CT) of the abdomen was arranged and this showed acute type B aortic dissection. By the time the CT was performed, the patient had been in hospital for 16 hours, almost 22 hours from the onset of pain.

## Introduction

Aortic dissection is a relatively uncommon catastrophic illness, the diagnosis of which has many potential pitfalls. Acute aortic dissection may mimic other common conditions, such as acute coronary syndrome (ACS), pulmonary embolism, heart failure, stroke, and acute abdomen; on the other hand, it can lead to these conditions depending on the location of the tear [1] so misdiagnosis of aortic dissection is not uncommon [2,3]. Because acute aortic dissection may be rapidly fatal, early recognition and emergency treatment are critical. Aortic dissection occurs when a tear of the intima in the aorta leads to a leak of blood with high pressure into the media, causing further separation of the two layers downstream with an incident rate of 5 to 30 cases per 1 million [1]. Acute aortic dissection presents with a wide range of manifestations of which chest and/or back pain is the most prevalent symptom. Other manifestations include abdominal pain, syncope, stroke, neurological deficit, pulse deficit, hypotension, normotension, hypertension, shock, cardiac tamponade, or limb ischaemia [1,4]. Classic findings are often absent, so a high clinical index of suspicion is necessary. Pain is often described as a tearing pain in the chest and/or back, localised or radiating to the neck or shoulders, and this is unlike the pain of coronary ischaemia, which can be of more gradual and less abrupt onset [4]. Pain has also been reported to be of sharp quality.

## Case presentation

A 77-year-old female presented with a sudden onset of chest and back pain for the last six hours. The pain was described as continuous, pleuritic, non-radiating, and localised to the front and back of the chest. The pain eased when the patient leaned in forward and backward movements. Pain severity was 8/10. She did not report shortness of breath, loss of consciousness, weakness, or diaphoresis but did report some nausea. She denied prior exertional or resting chest pain or a previous similar episode. Her medical history included hypertension, atrial fibrillation, pulmonary hypertension with right heart failure, thyroidectomy, diverticular disease, and a previous Hartman’s procedure for a perforated sigmoid diverticulum. She was taking levothyroxine 75 micrograms once daily, doxazocin 8 mg twice daily, diltiazem SR (slow release) 180 mg twice daily, losartan 100 mg once daily, furosemide 40 mg once daily, and rivaroxaban 20 mg once daily. Despite these conditions, she had been living independently and generally in good health.

On arrival, she was still in pain 6-7/10, afebrile, with a heart rate (HR) of 78 bpm, respiratory rate of 20/min, BP of 178/90, blood oxygen saturation of 98% on room air, and a repeat blood pressure showed no significant discrepancy in reading in both arms (207/106 mm of Hg on the right arm and 205/96 on the left arm). Physical examination revealed chest wall and back tenderness and mild abdominal discomfort on palpation, which the patient claims have been longstanding since Hartman's surgery, normal vesicular breathing, and heart sounds were unremarkable with no added sounds on auscultation. 

The differential diagnoses at this point were acute coronary syndrome (ACS), aortic dissection, and musculoskeletal pain secondary to vertebral fracture or collapse.

**Figure 1:**
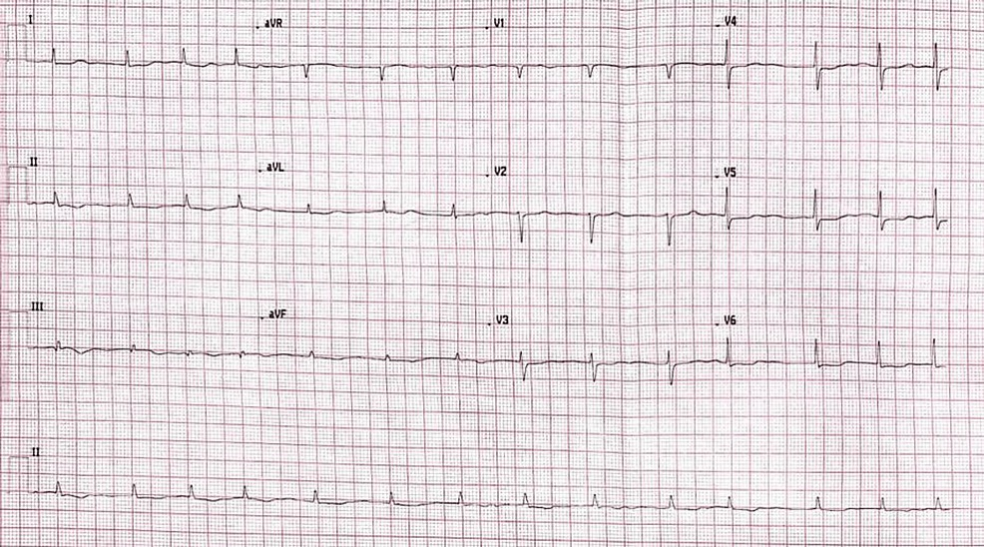
ECG showing AF with subtle inverted T waves in leads II, III, aVF, and mild ST segment depression in leads V4-V6 ECG; electrocardiogram, ACS; acute coronary syndrome

**Figure 2:**
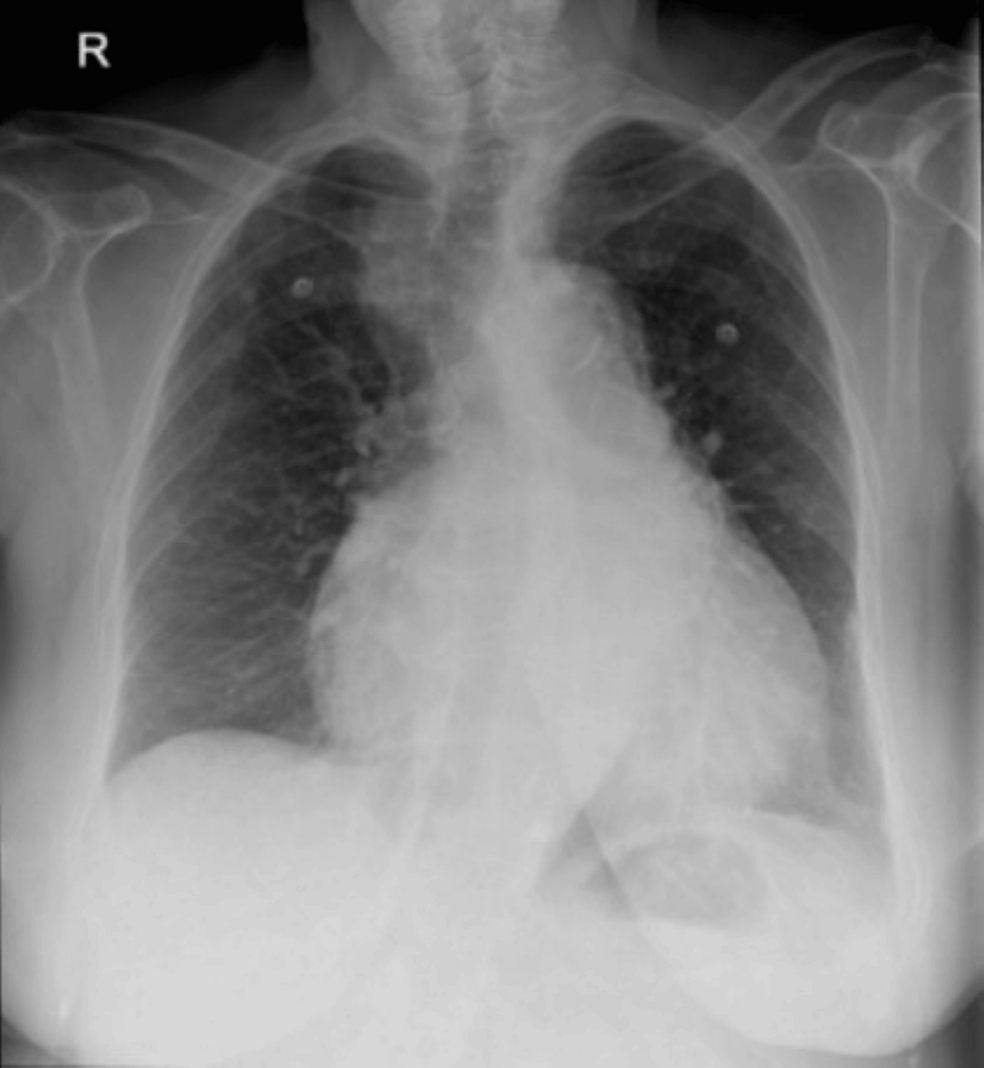
Chest X-ray Chest X-ray reveals normal mediastinum width and cardiomegaly; the patient was known to have cardiomegaly.

**Figure 3:**
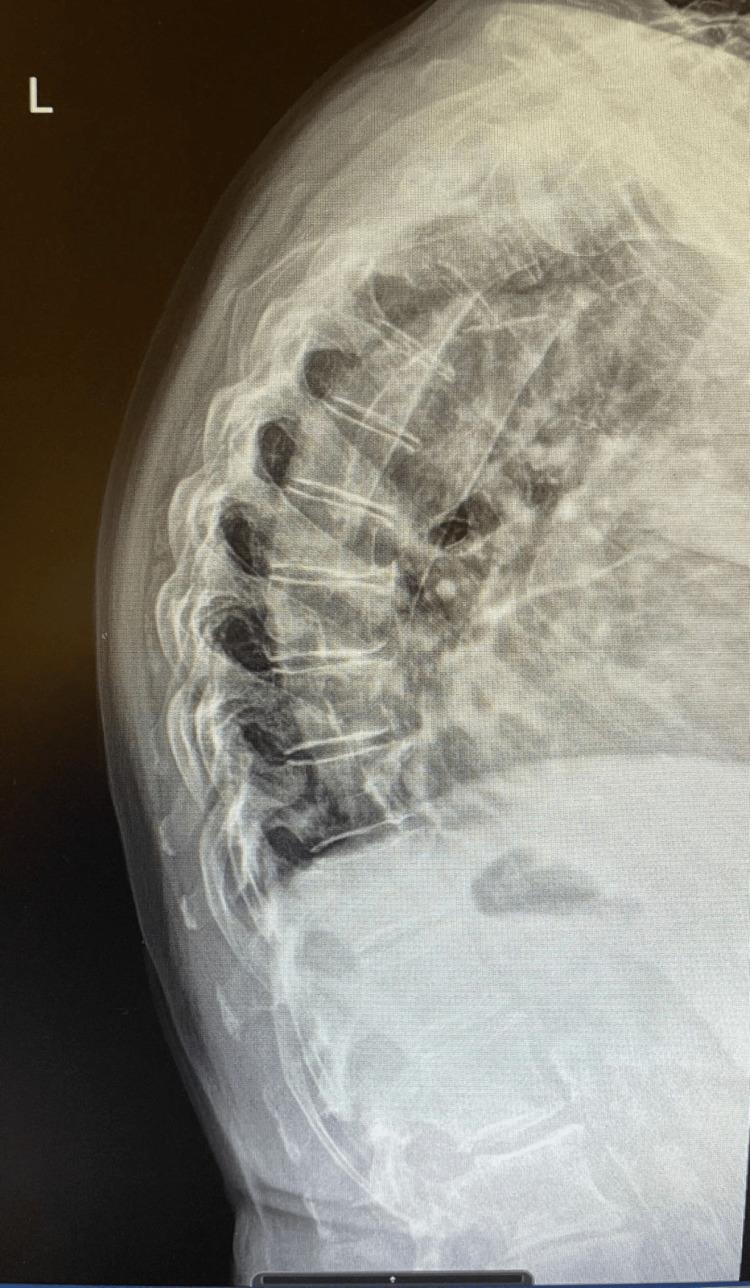
Thoracic spine X-ray lateral view Lateral view thoracic spine X-ray shows mild to moderate generalised degenerative changes with no evidence of vertebral collapse.

Initial blood tests, including venous lactate, were unremarkable, apart from a mildly raised Troponin-I of 68 ng/L (normal value 0-0.04) (Table 1). The electrocardiogram (Figure 1) showed the known atrial fibrillation with slightly new inverted T waves in leads II, III, and aVF, and mild ST segment depression in leads V4-V6 leads. Chest radiograph revealed known cardiomegaly with no features of mediastinal widening (Figure 2). The dorsal spine radiograph ruled out vertebral fracture or collapse (Figure 3).

**Table 1:** Summary of the patient's laboratory data on admission showing a less than 20ng/L change in troponin I, but otherwise no significant deranged values ALT, alanine aminotransferase; ALP, alkaline phosphatase; WBC, white blood count; PLT, platelets; RBC, red cell count; MCV, mean corpuscular volume; Hb, haemoglobin; INR, international normalised ratio; APTT; activated partial thromboplastin time; CRP, C-reactive protein *Local trust protocol: if baseline troponin I is less than 3 ng/L or change at 2 hours is less than 20 ng/ l then myocardial infarction is unlikely.

Test	Result	Reference range
Sodium	142.2 mmol/L	133-146 mmol/L
Potassium	4.0 mmol/L	3.5-5.2 mmol/L
Urea	8.9 mmol/L	2.7-7.9 mmol/L
Creatinine	80 umol/L	44-71 umol/L
ALT	13 U/L	10-49 U/L
ALP	73 U/L	30-130 U/L
Bilirubin	12 umol/L	<21 umol/L
Albumin	45 g/L	35-50 g/L
Total Protein	73 g/L	60-80 g/L
Amylase	40 IU/L	30-110 IU/L
Calcium	2.36 mmol/L	2.20-2.60 mmol/L
Adjusted Calcium	2.26 mmol/L	2.20-2.60 mmol/L
WBC	9.50 x10^9/L	4.00-11.00 x10^9/L
Neutrophil	8.17 x10^9/L (H)	2.00-7.00 x10^9/L
Lymphocytes	0.70 x10^9/L (L)	1.00-3.00 x10^9/L
PLT	233 x10^9/L	140-400 x10^9/L
RBC	4.46 x10^12/L	4.10-5.10 x10^12/L
MCV	94.4 fL	82.0-105.0 fL
Hb	131 g/L	120-150 g/L
Prothrombin time	29.1 seconds (H)	12.0-16.0 seconds
INR	1.4 (H)	0.8-1.2
APTT ratio	1.9 (H)	0.8-1.2
CRP	<4 mg/L	<10 mg/L
Initial troponin I	68 ng/L	0-0.04 ng/L *
Repeat troponin I ( 2 hours )	76 ng/L	0-0.04 ng/L *

**Figure 4:**
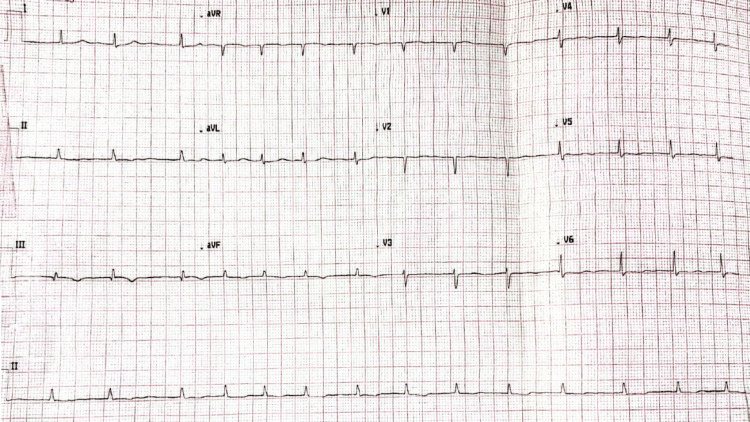
Repeat ECG showing no acute dynamic changes despite ongoing chest pain to support the diagnosis of ACS ECG; electrocardiogram, ACS; acute coronary syndrome

Based on the above assessment, the diagnosis was narrowed to ACS. A repeat ECG didn’t show any dynamic changes (Figure 4) and a second set of troponin I showed a slightly raised level at 78 ng/L (< 20 ng/L rise). The patient was reviewed by the cardiology team who deemed her symptoms were unlikely to be cardiac due to the nature of the pain being of sudden onset, worse on inspiration, reproducible on palpation, with no significant change in troponin and absence of dynamic ECG changes. Hence, they recommended continuing rivaroxaban.

Further assessment of the patient

Our patient was reassessed and by this time, her pain improved with morphine sulphate to 4-5/10 with ongoing chest wall and back tenderness. It was noted that the abdomen was more tender on very mild palpation and the tenderness was more marked at the epigastric region although it remained soft and lax with no guarding or rigidity and normal bowel sounds on auscultation; however, she reported passing wind normally. Considering the previous history of the perforated diverticulum, an urgent computed tomography (CT) scan of the abdomen and pelvis with contrast was arranged.

Her blood pressure remained elevated at 173/90 despite receiving blood pressure medications, and this was attributed to the pain the patient was experiencing.

**Figure 5:**
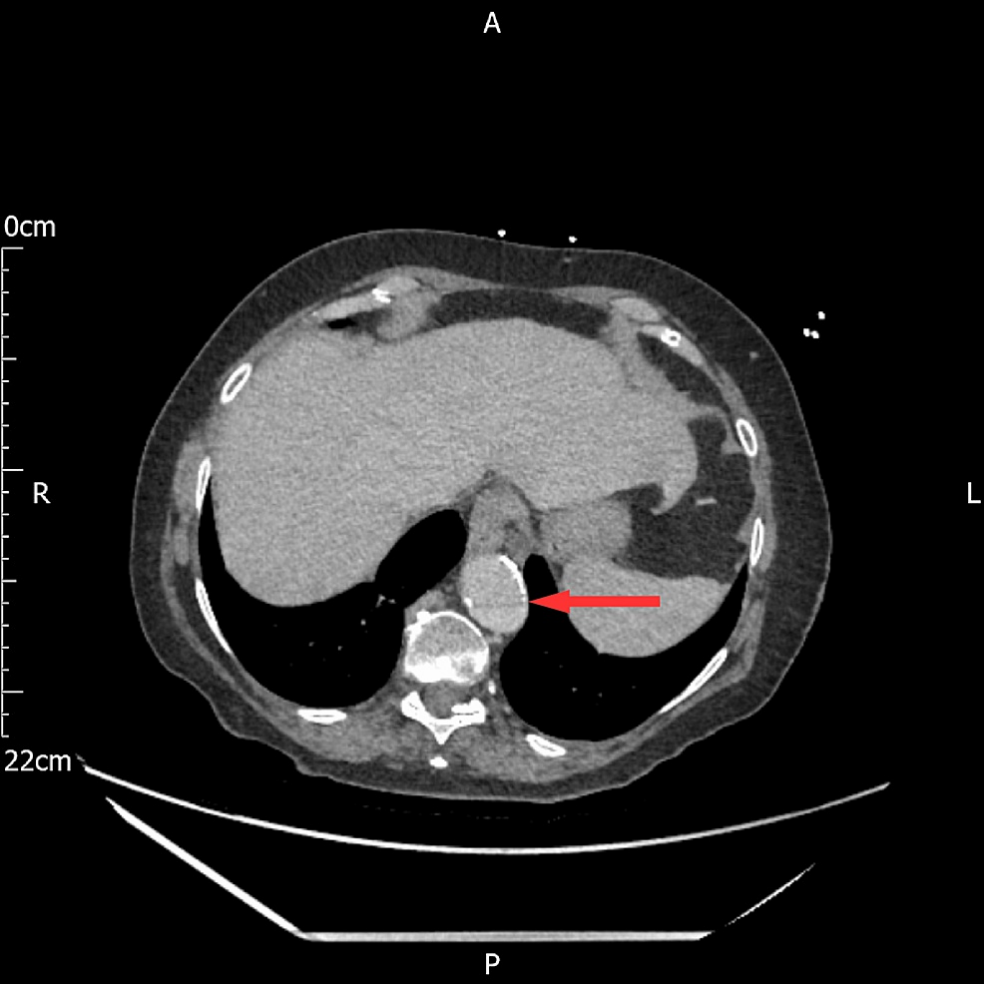
Portal venous phase CT AP showing aortic dissection at the T 10 level CT AP; computed tomography of abdomen and pelvis, T; thoracic

**Figure 6:**
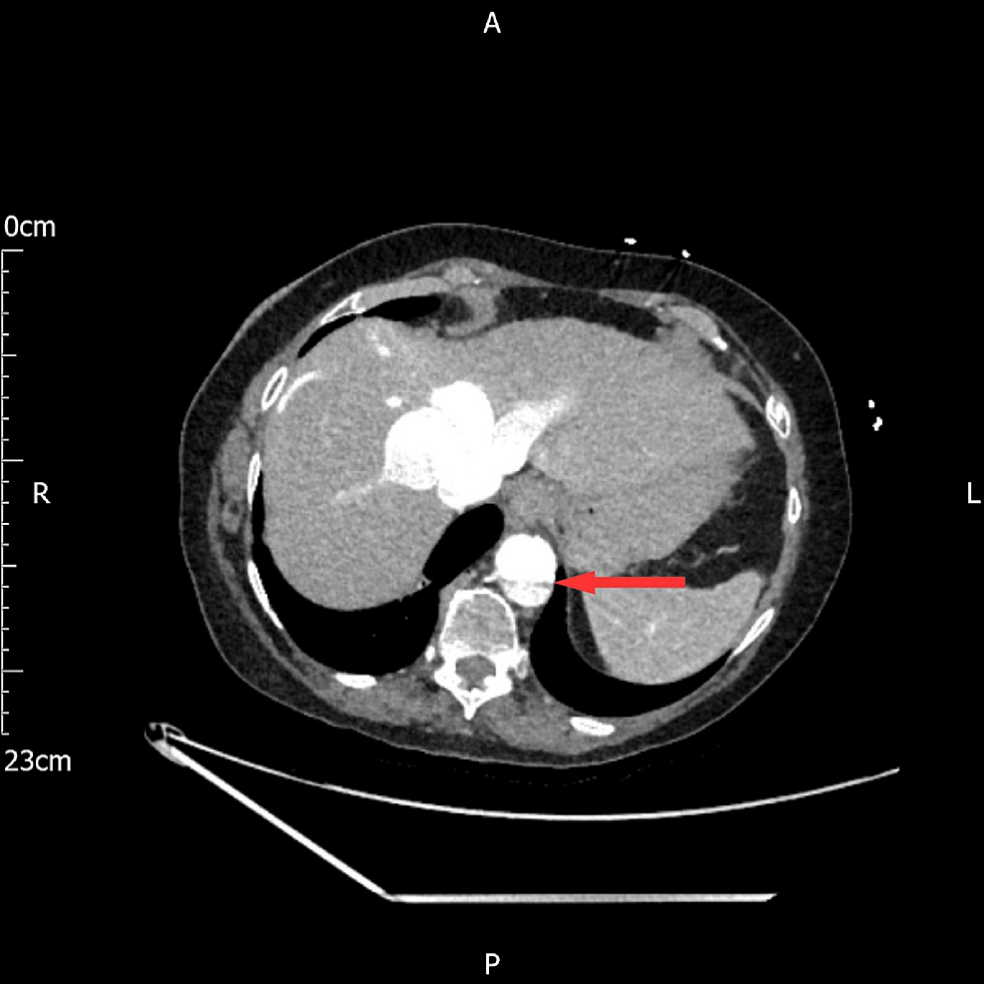
Arterial phase CTA showing aortic dissection at the T 10 level CTA; computed tomography angiogram, T; thoracic

**Figure 7:**
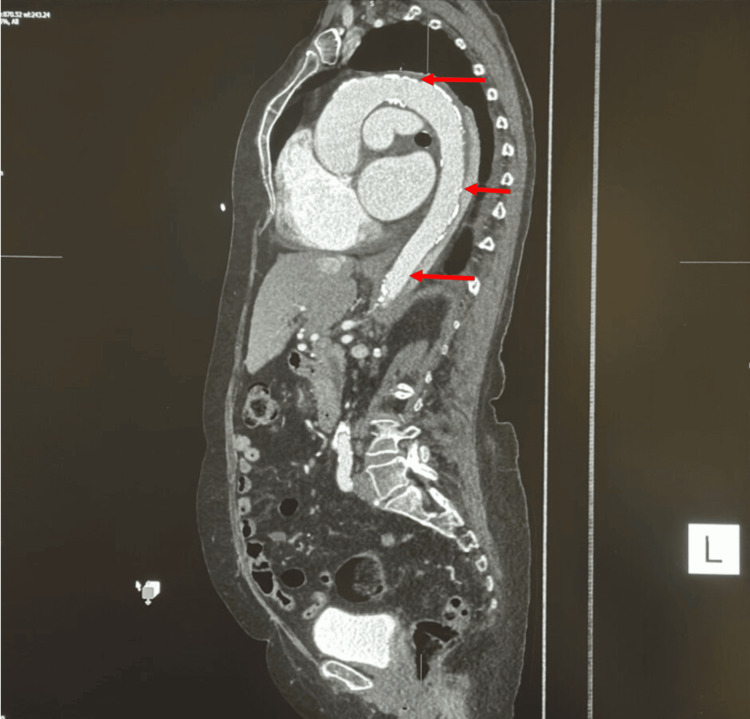
Sagittal section arterial phase angiogram showing a Stanford Type B/DeBakey III dissection with the dissection flap (red arrows)

An urgent call was received from the radiology department stating that there was a dissection identified in the partially imaged descending thoracic aorta on the portal venous contrast-enhanced CT scan (Figure 5), which was presumed to be the cause of the patient's epigastric pain. A dedicated arterial phase contrast-enhanced CT scan of the thoracic aorta confirmed Type B aortic dissection (Figure 5) as per the Stanford classification and type III as per the DeBakey classification (Figures 6, 7). By the time the CT was performed, the patient had been in hospital for 16 hours, almost 22 hours from the onset of the pain. This delay could have led to fatal consequences, such as rapid aortic expansion, malperfusion, and aortic rupture, leading to internal bleeding and death.

The patient was reviewed by vascular surgeons who advised conservative management focusing on blood pressure control, heart rate monitoring, and pain control along with regular monitoring of any arising aortic dissection complication. She received glyceryl trinitrate (GTN) infusion and metoprolol. Her presentation was discussed at the vascular MDT (multidisciplinary meeting) and it was planned to monitor the dissection radiologically and clinically and to intervene surgically in case of progression of dissection or if the pain did not settle. She responded to medical management and the dissection remained stable on monitoring CT scans of the aorta avoiding any surgical intervention. It took a few days to titrate off the GTN infusion, achieve the target blood pressure of less than 120/80, and introduce an oral antihypertensive. She was discharged two weeks later on amlodipine 10 mg once daily, bisoprolol 5 mg twice daily, doxazocin 8 mg twice daily, hydralazine 50 mg three times per day, losartan 100 mg once daily, and spironolactone 12.5 mg once daily. Following discharge, the dissection was monitored radiologically at one, six, and 12 months, which showed no further progression in the dissection. The patient is currently awaiting a follow-up scan at a 12-month interval.

Two months later, she presented with postural dizziness and low BP and adjustments were made aiming to keep her BP target at around 120/80 mmHg.

## Discussion

Two systems exist for the classification of aortic dissections, and these help guide further management of patients. In the Stanford classification, type A aortic dissection involves the ascending aorta, whilst in Type B, the ascending aorta is spared. DeBakey type I involves the ascending aorta, aortic arch, and descending aorta. Dissection confined to the ascending aorta is DeBakey type II while that which only involves the descending aorta distal to the origin of the left subclavian artery is the DeBakey III dissection. Patients with DeBakey type I and type II or Stanford type A require emergency cardiac surgery as they are at risk of life-threatening complications. On the other hand, in patients with uncomplicated Stanford type B or DeBakey III dissection medical therapy is preferred initially but may require surgical intervention in case of evolving complications such as imminent rupture, expansion, shock, or mal-perfusion syndromes [4]. Patients with type B dissection more often experience pain in the back or abdomen [5].

The present case highlights the challenges of suspecting and diagnosing acute aortic dissection in a timely manner so that time-sensitive interventions are made, as the symptoms were overlapping between ACS and acute abdomen pain, causing a diagnostic dilemma. Aortic dissection was not suspected due to the absence of mediastinal widening on the chest X-ray and no blood pressure discrepancy in both arms and later the diagnosis was made incidentally on the abdominal CT performed for a suspected perforated viscous. The character of pain being abrupt and sharp, and the elevated blood pressure should have prompted the clinician to consider and investigate for aortic dissection when ACS was ruled out. Up to 30% of patients later found to have aortic dissection were initially suspected of having other conditions such as ACS, aneurysms, pericarditis, pulmonary embolism, aortic stenosis, or even cholecystitis [1,6].

The differential diagnosis of ACS may be of particular importance because thrombolytic therapy may be fatal in aortic dissection leading not only to delayed diagnosis of the aortic dissection, but risk of inappropriate treatment with antiplatelet, antithrombin, and fibrinolytic therapies and exposure to these agents is associated with increased bleeding risk and increased mortality [7]. Being a medical emergency, aortic dissection requires urgent diagnosis and prompt treatment, as the mortality rate is as high as 1% per hour during the first 48 hours after the onset of symptoms if left untreated. In this case, it took 22 hours to reach the final diagnosis; hence, the patient had a 22% mortality rate.

Patients typically present with abrupt onset of severe chest, back, or abdominal pain that is described as sharp or stabbing. Anterior chest pain is typical in patients with type A dissection and can be localised or follow the path of the dissection, whereas abdominal and back pain are more commonly associated with type B dissection though there is overlap as in our case. Other symptoms to consider are shortness of breath, syncope, stroke symptoms, heart failure symptoms, paraplegia, or symptoms of mesenteric and peripheral ischaemia [4]. Silent painless aortic dissection has also been reported as well as chronic abdominal pain [8]. Classical physical signs include elevated blood pressure, blood pressure discrepancies between the upper limbs, pulse deficit, diastolic murmur of aortic regurgitation, and heart block on ECG. Other physical signs include hypotension, focal neurology, and symptoms of peripheral ischaemia such as limb numbness, absent peripheral pulses, and cold extremities.

Hypertension, age, atherosclerosis, prior aortic surgeries, and connective tissue disorders, such as Marfan, Ehlers-Danlos type 4, Turner syndrome, and familial thoracic aortic aneurysm are the most common risk factors predisposing to the weakness of the aortic wall, leading to acute aortic dissection, with 72% of patients reported to be hypertensive [1,5]. Other risk factors include cocaine or other stimulant use, weightlifting, trauma, and coarctation of the aorta. The diagnosis should also be considered in young patients even if they have no risk factors.

## Conclusions

Aortic dissection can be often mistaken for cardiac, abdominal, muscular, or neurological disease, leading to a high mortality rate due to delays in diagnosis and management. This case highlights the significance of familiarising with the variable presentation of acute aortic dissection and the challenges it may pose for prompt diagnosis. A normal chest radiograph or absence of blood pressure discrepancies doesn't rule out the possibility of this condition, and these signs are also non-specific. Clinicians should exercise a high level of suspicion and organise appropriate radiological investigations (contrast-enhanced CT scans) to confirm or refute this emergency condition.
